# Is the plantaris muscle the most undefined human skeletal muscle?

**DOI:** 10.1007/s12565-020-00586-4

**Published:** 2020-11-07

**Authors:** K. Kurtys, B. Gonera, Ł. Olewnik, P. Karauda, R. Shane Tubbs, M. Polguj

**Affiliations:** 1 Department of Anatomical Dissection and Donation, Medical University of Lodz, Żeligowskiego 7/9, 90-136 Łódź, Poland; 2 Department of Normal and Clinical Anatomy, Medical University of Lodz, Żeligowskiego 7/9, Łódź, 90-136 Poland; 3 Department of Neurosurgery, Tulane University School of Medicine, New Orleans, LA USA; 4 Department of Neurosurgery and Ochsner Neuroscience Institute, Ochsner Health System, New Orleans, LA USA; 5 Department of Anatomical Sciences, St. George’s University, St. George’s, Grenada

**Keywords:** Case report, Knee, Knee joint, Plantaris muscle, Plantaris muscle origin

## Abstract

The plantaris muscle is located in the posterior aspect of the superficial compartment of the lower leg, running from the lateral condyle of the femur to the calcaneal tuberosity. Classically, it is characterized by a small and fusiform muscle belly, which then changes into a long slender tendon. From the evolutionary point of view, the muscle is considered vestigial. However, it has recently been suspected of being a highly specialized sensory muscle because of its high density of muscle spindles. It has a noticeable tendency to vary in respect of both origin and insertion. Researchers have published many reports on the potential clinical significance of the muscle belly and tendon, including mid-portion Achilles tendinopathy, ‘tennis leg syndrome’, and popliteal artery entrapment syndrome. The right knee joint area was subjected to classical anatomical dissection, during which an atypical plantaris muscle was found and examined in detail. Accurate morphometric measurements were made. The muscle belly was assessed as bifurcated. Morphologically, superior and inferior parts were presented. There was a tendinous connection (named band A) with the iliotibial tract and an additional insertion (named band B) to the semimembranosus tendon. Both bands A and B presented very broad fan-shaped attachments. The human plantaris muscle is of considerable interest and has frequent morphological variations in its proximal part. Its specific characteristics can cause clinical problems and lead to confusion in diagnosis. More studies are needed to define its actual features and functions.

## Introduction

The plantaris muscle (PM) belongs to the posterior superficial compartment of the lower leg. It has origin in the posterior aspect of the knee joint. Normally, its single, small and fusiform muscle belly originates on the lateral supracondylar line of the femur, superior and medial to the lateral head of the gastrocnemius muscle and to the knee joint capsule. It develops into a long thin tendon descending along the lower leg into a space between the gastrocnemius and soleus muscle. Ultimately, the plantaris tendon reaches a distal attachment, the calcaneal tuberosity (Spina 2007; Moore and Dalley 2008; Olewnik et al. 2017b, 2018b; Vlaic et al. 2019).

Muscles, tendons, ligaments or vessels are well known to be morphologically variable, but some have a particular tendency to vary (Olewnik et al. 2017a,2018a,2019a,2020a; b, c). The PM shows considerable morphological variability in both its proximal and distal attachments and even its course (Olewnik et al. 2017b, 2020a). Besides the classified variations named *types*, there are also some very rare cases (Gonera et al. 2020; Kurtys et al. 2020). Because of their rarity, they could seem insignificant, but clinical science shows that even uncommon variants are sometimes crucial for correct differentiation and diagnosis and, therefore, for the health of patients (Rohilla et al. 2013).

Some anatomical, radiological and surgical studies demonstrate that the PM can cause medical problems, affect their development negatively or lead to difficulties in diagnosis (van Sterkenburg et al. 2011a; b; Alfredson 2011; Rohilla et al. 2013; Spang et al. 2013; Olewnik et al. 2017b; Alfredson and Spang 2017). The main focus for many years has been on the plantaris tendon and its distal attachment. However, scientists have recently noticed that the proximal attachment of the PM is as highly variable as the distal one and can also prove clinically significant (Ahmed et al. 2017; Joshi et al. 2014; Olewnik et al. 2020a). The proximal part of the PM is susceptible to a range of possible injuries, both the muscle belly and the tendon potentially being ruptured at the muscle–tendon junction; such injuries have been classified as ‘tennis leg’ (Spina 2007; Rohilla et al. 2013; Vlaic et al. 2019; Olewnik et al. 2020a). It is possible that rupture of the bifurcated variant of the PM, such as presented one in this publication, may provide clinicians with more difficulties in distinguishing and diagnosing a PM rupture and so ‘tennis leg’ as well.

This study presents a description of a bifurcated PM, characterized by atypical connections with the semimembranosus muscle and the iliotibial tract. In our opinion, the complex, proximal part of the PM can create problems for clinicians, including surgeons and orthopedists. The present case report was written to alert them to this particular sort of PM.

## Case report

Classical anatomical dissection of the right knee joint was strictly planned as an educational demonstration for medical students at the Department of Anatomical Dissection and Donation, Medical University of Lodz. A 71-year-old male cadaver was dissected. Standard techniques were used to investigate that anatomical area in accordance with a detailed specified protocol (Olewnik et al. 2018a, b, 2019a,2020a).

**Fig. 1:**
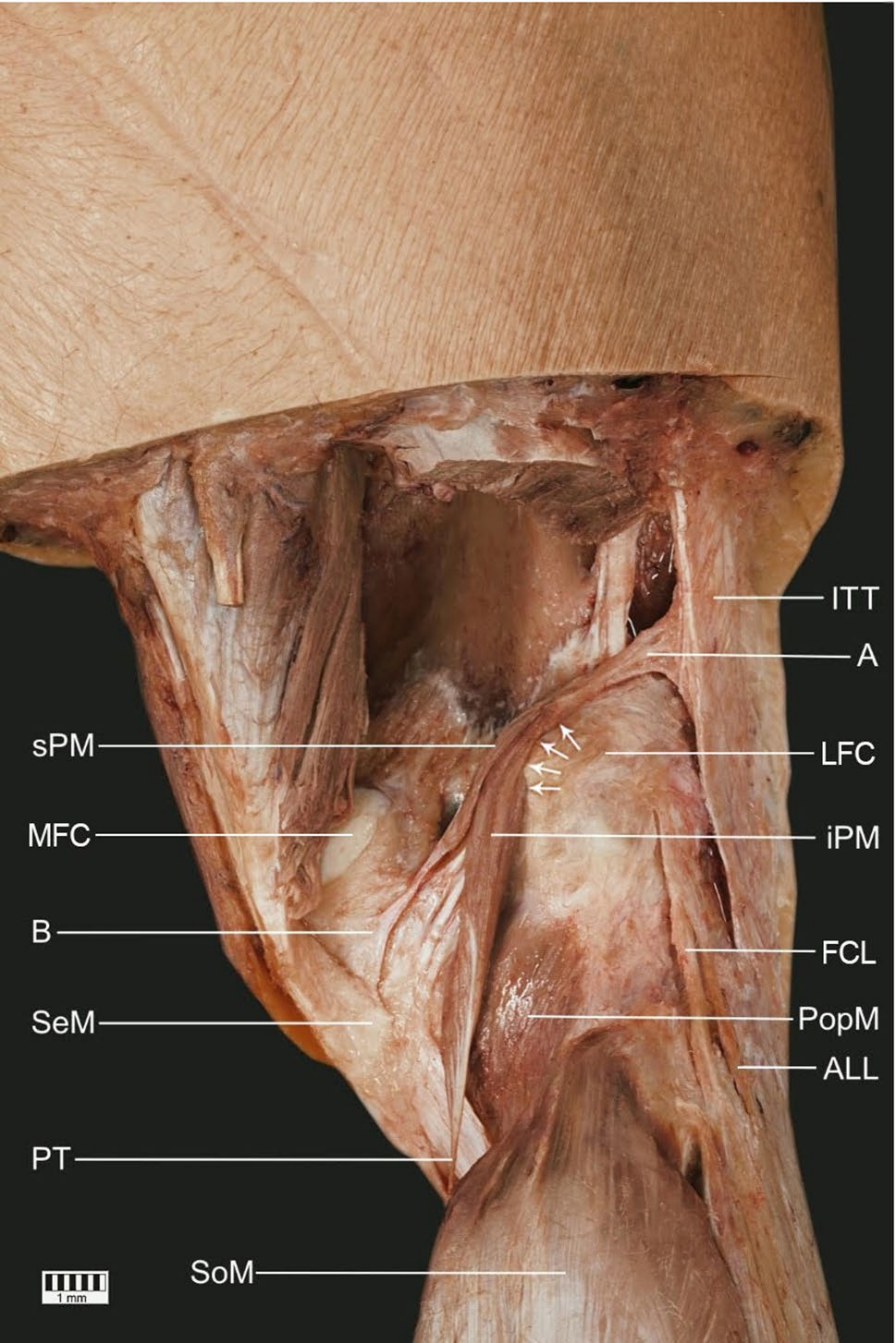
The presented variant of the plantaris muscle. Posterolateral view of the right knee joint. *ITT* the iliotibial tract, *A* the tendinous band between the plantaris muscle and the iliotibial tract, *sPM* the superior part of the plantaris muscle, *iPM* the inferior part of the plantaris muscle, *B* the additional insertion to the semimembranosus tendon, *FCL* the fibular collateral ligament, *PopM* the popliteus muscle, *SeM* the semimembranosus muscle (tendon), *ALL* the anterolateral ligament, *PT* the plantaris tendon, *SoM* the soleus muscle, *LFC* the lateral femoral condyle, *MFC* the medial femoral condyle, *arrows* indicate an attachment to the lateral femoral condyle

**Fig. 2:**
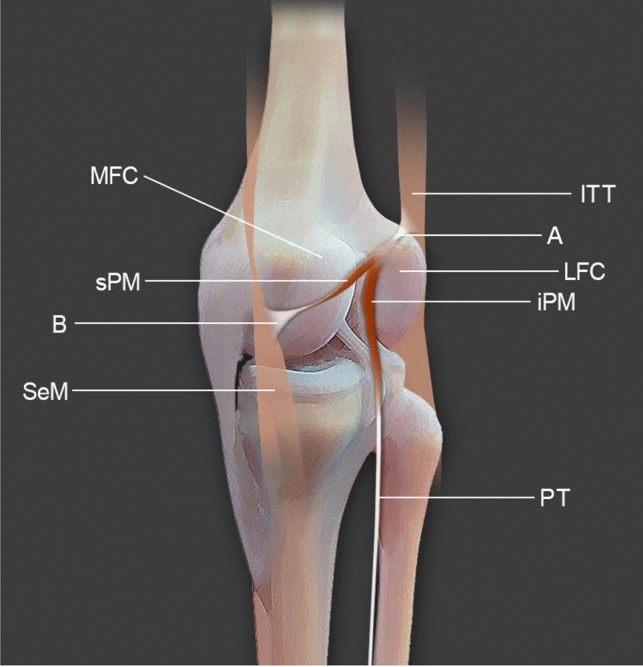
A schema of the presented variant of the plantaris muscle. Posteromedial view of the right knee joint. *ITT* the iliotibial tract, *A* the tendinous band between the plantaris muscle and the iliotibial tract, *sPM* the superior part of the plantaris muscle, *iPM* the inferior part of the plantaris muscle, *B* the additional insertion to the semimembranosus tendon, *SeM* the semimembranosus muscle (tendon), *PT* the plantaris tendon, *LFC* the lateral femoral condyle, *MFC* the medial femoral condyle

During the dissection, the plantaris muscle was distinguished. After the more detailed investigation of this muscle, its unusual features were noticed and assessed as worthy of publication. There was a bifurcated muscle belly: superior and inferior parts. Both parts originated similarly from the lateral femoral condyle and the iliotibial tract with a specific tendinous band (band A; Figs. 1, 2). Their muscle fibers were firmly connected and inseparable in its initial part. The inferior part ran downwards and developed into the classical long and slender plantaris tendon. Band A was relatively broad and its attachment to the iliotibial tract was fan-shaped. Although the superior part presented the same origin as the inferior one, it had significantly less muscle mass. That part was directed more horizontally and then became a short thin tendon (band B) running towards the semimembranosus tendon, which it reached with a very broad, fan-shaped attachment (Figs. 1, 2). One muscular branch from the tibial nerve ran towards the plantaris muscle and divided into two small branches (one for each part: superior and inferior) just before entering into the muscle. There were no abnormalities around the tibial nerve and popliteal vessels.

Following the morphological examination of this unusual plantaris muscle, measurements were made. These were taken as digital photographic images and processed through MultiScanBase 18.03 (Computer Scanning System II, Warsaw, Poland) (Gonera et al. 2020; Kurtys et al. 2020). All of them are presented in Table 1.

**Table 1:** Presentation of collected measurements

	Length (mm)	Width in the widest point (mm)	Width in the origin/middle/myotendinous junction (mm)	Width in the myotendinous junction/middle/insertion (mm)
The superior part	31.32	3.05	–	–
The inferior part	72.49	8.16	–	–
Band A	23.38	–	23.26/4.12/4.49	–
Band B	26.09	–	–	2.22/1.98/22.44

## Discussion

Evolutionary science has characterized many anatomical structures in terms of origin, development, or function, though some remain unclear (Cruveilhier 1834; Lewis 1902; Sambasivan et al. 2011; Ericsson et al. 2013; Capdarest-Arest et al. 2014; Laitman 2018; D’Elia and Dasen 2018; Diogo et al. 2019). Originally, the plantar aponeurosis was the point of insertion of the plantaris tendon. Subsequently, the PM changed its primary distal attachment because of the evolutionary change in human body posture, so it reached the calcaneal tuberosity (Cruveilhier 1834). The PM is considered vestigial because it is small and biomechanically insufficient regarding knee joint flexion and ankle joint plantar flexion (Menton 2000; Vlaic et al. 2019). However, it is reported to have a proprioceptive rather than mechanical function because of its small muscle belly, long, slender tendon and high density of muscle spindles (Menton 2000). Interestingly, the muscle spindles are considerably more dense in the PM than in the gastrocnemius and soleus muscles; 3.7 spindles per gram in the PM and 0.67 spindles per gram in the other two (Peck et al. 1984; Vlaic et al. 2019). It can be asked whether the PM is a disappearing muscle or undergoing evolutionary change to a new highly specialized sensory muscle. We believe that such an anomalous plantaris muscle like presented one in this study may be an indication that it is not going to vanish but evolve instead.

Most studies show the PM not to be a constant muscle (7–20% absence) [1,39–42], though two publications reported its presence in 100% (Aragão et al. 2010; van Sterkenburg et al. 2011a). Anatomical classifications have been created to distinguish its most frequent types of origin (Ahmed et al. 2017; Olewnik et al. 2020a). Olewnik et al. (2020a) presented the most recent and elaborate, sixfold classification system; the first five types are classic variants, while all ‘rare cases’ are categorized as Type VI (Table 2). Types I–V have single muscle bellies and the most interesting amongst them seemed to be Type IV (prevalence – 6.3%), characterized by a tendinous connection to the iliotibial tract. The first ‘rare case’ (Type VI) depicted in this study featured two muscle bellies connected separately to the iliotibial tract. The additional head fused with the oblique popliteal ligament and together with it reached the semimembranosus tendon. The second “rare case” was less complex and was bifurcated. Two other publications reported noteworthy types of origin of the PM (Freeman et al. 2008; Nayak et al. 2010). One found a variant with a connection to the fibular collateral ligament (prevalence – 13.46%) (Nayak et al. 2010), and the other described a fibrous bridge between the PM and the patella (prevalence – 10.9%) (Freeman et al. 2008). Such types have been described nowhere else.

**Table 2:** Classification of PM origins according to Olewnik et al. (2020a, b)

A type of the PM origin	Prevalence (%)
Type I	
Subtype A: the lateral head of the gastrocnemius muscle, lateral condyle of the femur, knee joint capsule	40.5
Subtype B: the lateral head of the gastrocnemius muscle, lateral condyle of the femur, knee joint capsule, popliteal surface of the femur	8.7
Type II: the knee joint capsule and lateral head of the gastrocnemius muscle	25.4
Type III: the lateral condyle of the femur and knee joint capsule	10.3
Type IV: the lateral femoral condyle, knee joint capsule and iliotibial tract	6.3
Type V: the lateral condyle of the femur	8.8
Type VI: ‘rare cases’	
Double PM:	1 case
The main head: the lateral femoral condyle, knee joint capsule, iliotibial tract	
The accessory head: the iliotibial tract	
Bifurcated PM:	1 case
The lateral head: the lateral head of the gastrocnemius muscle	
The medial head: the knee joint capsule	
The presenting case (bifurcated PM):	1 case
The superior part: the iliotibial tract, lateral condyle of the femur	
The inferior part: the iliotibial tract, lateral condyle of the femur	

Besides original articles concerning the origin of the PM, there are also interesting case reports confirming its strong tendency towards anatomical variability. According to Kotian et al. (2013), a bifurcated PM is possible. It arose from the lateral condyle of the femur as a single muscle. After one centimeter, a split was noticed, and two separate muscular entities (superior and inferior) were revealed. Tendinous fibers from the superior head inserted mainly on the knee joint capsule around the medial condyle of the femur. However, a thin tendinous band started descending from the insertion point to merge with a long slender tendon from the inferior head. This study emphasized the correlation between the two heads, nerves and vessels running through the popliteal fossa. Another interesting finding was a unilateral PM in an adult male cadaver with an extremely small muscle belly (2 cm length and 0.5 cm width) and no normal plantaris tendon. Its origin was classical but the distal attachment was a thin fascia, owing to which it merged with the soleus muscle close to its origin (Sugavasi 2013). The most recent anatomical finding concerning PM origins is a remarkable three-headed example. The first and second heads each originated from two places; the first from the posterior femoral surface and the lateral femoral condyle, the second from the lateral femoral condyle and the lateral head of the gastrocnemius muscle. The third head had only one origin, namely the lateral head of the gastrocnemius muscle. Each muscle belly possessed its own tendon, and these connected to each other to form a common band (Olewnik et al. 2020b).

To some extent, the variant origin of the PM described in the present study resembles those previously reported, but it is still exceptional and merits direct attention. The muscle was bifurcated and two muscle bellies, superior and inferior, were distinguished. Kotian et al. (2013) also described a bifurcated PM, originating from the lateral condyle of the femur, but there was no really interesting tendinous connection between it and the iliotibial tract. Furthermore, the superior head in their study seemed more massive than the inferior; the opposite applied in our case. A tendinous connection between the muscle and the iliotibial tract was also noticed in one study (Olewnik et al. 2020a). A merger of this sort featured in the double variant of the PM, but the two muscle bellies attached to it separately. In contrast to that study, the connection in our case was single and the attachment from the iliotibial tract side was fan-shaped. Two muscle parts (superior and inferior) originated together from the tendinous band and the lateral condyle of the femur and then went towards their own insertions. The inferior one appeared to be the classical PM, descending at a slant medially and developing into the long thin plantaris tendon. The latter (superior), with a shorter and thinner muscle belly, headed more transversely to the medial side of the knee joint and merged with the semimembranosus tendon with a relatively thin tendinous band, creating the fan-shaped insertion at the end. Interestingly, no fusion was found between the tendon of the superior part and the oblique popliteal ligament, in contrast to the abovementioned double PM. Nevertheless, scientists could consider whether such spare muscle bellies of the PM, running throughout the width of the knee joint, could work as additional stabilizers of the posterior aspect of the knee joint.

Until recently, most researchers were more interested in the plantaris tendon than the PM belly. However, the variability around the muscle belly is now becoming clinically interesting as well (Olewnik et al. 2020a). The problem of “tennis leg” has received increasing attention in recent years. There is disagreement among scientists as to whether a PM injury (such as a rupture) should be classed as one of causes of this condition (Delgado et al. 2002; Spina 2007; Rohilla et al. 2013). Typically, “tennis leg” can develop when the knee joint is entirely extended and the ankle joint is in dorsiflexion. In some athletes, this arrangement of the lower extremity results in passive straining of the muscle and rupturing can result (Kwak et al. 2006). According to some opinions, ‘tennis leg’ concerns only a rupture or tear of the medial head of the gastrocnemius muscle (Arner and Lindholm 1958; Miller 1977; Delgado et al. 2002). Doubtless such an injury is the most common cause of this problem, as shown by Delgado et al. (Delgado et al. 2002); it was the most frequently diagnosed cause amongst patients presenting symptoms of ‘tennis leg’ (prevalence 66.7%). Nevertheless, although PM rupture was reported as the cause in far fewer cases (1.4%) in that ultrasound study, it still can be a cause, albeit rarely. The other findings triggering clinical symptoms of ‘tennis leg’ in 141 patients are shown in Table 3. Rohilla et al. (2013) propose that the definition should be broadened to include rupture of the PM and the semimembranosus tendon or their muscle bellies. We also believe that it is necessary to extend the definition of ‘tennis leg’ to include subtypes of ruptures/tears of the PM or the semimembranosus muscle. In our opinion, rupture of the bifurcated variant of the PM presented in this study could be more difficult to distinguish and diagnose as a PM rupture.

**Table 3:** Ultrasound findings amongst patients (141) clinically diagnosed as presenting ‘tennis leg’ according to Delgado et al. (2002)

Ultrasound outcome	Prevalence (%)
Partial rupture of the medial head of the gastrocnemius muscle around the myotendinous junction	66.7
Fluid accumulation between aponeurosis of the gastrocnemius (medial head) and soleus muscle with no indication of muscle rupture	21.3
Deep vein thrombosis	9.9
Entire rupture of the plantaris tendon	1.4
Partial rupture of the soleus muscle	0.7

A few studies indicate that clinicians should remember the existence of the PM and its ability to vary so that certain medical problems around the knee joint are not misdiagnosed (Spina 2007; Rohilla et al. 2013; Vlaic et al. 2019; Olewnik et al. 2020a). According to Rohilla et al. (2013), a PM rupture was mistaken for deep vein thrombosis during an ultrasound examination. An appropriate distinction between these two conditions is important because their treatment approaches are entirely different. Besides deep vein thrombosis, a PM rupture can a mimic calf neoplasm or a ruptured Baker’s cyst (Spina 2007; Rohilla et al. 2013). A PM with two heads, separate or bifurcated, could cause still greater confusion in diagnosis. It seems it need not rupture to simulate a Baker’s cyst or a tumor; in our view, hypertrophy of the additional part/head could mislead similarly. Furthermore, even if a PM rupture usually heals on its own, it can become more serious when it is accompanied by bleeding and swelling. In such a case, an urgent fasciotomy is necessary (Rohilla et al. 2013).

Another clinical problem potentially involving the PM is PAES. This can occur when hypertrophied calf muscles press on the popliteal artery, decreasing blood flow to the lower leg and foot (Kwon et al. 2015; Olewnik et al. 2018c). An additional part/head of the PM could increase the risk for this condition, which is why clinicians should be informed about different variants of this muscle. Moreover, the correlation between the PM heads and the main vascular trunks running through the popliteal fossa could be relevant to the occurrence of this syndrome (Kotian et al. 2013; Olewnik et al. 2018c). According to Olewnik et al., it is also possible for the tibial nerve or its branch to be entrapped by the PM (Olewnik et al. 2018c, 2020a). They suggested the possibility that a PM with a connection to the iliotibial tract of the kind described in the present study could contribute to iliotibial band syndrome, which seems really interesting (Olewnik et al. 2020a). This condition most frequently concerns endurance athletes, in whom there are high-frequency repetition of movements of the knee joint (flexion and extension). The sign of this syndrome is a sharp burning pain on the lateral knee side and tenderness during physical examination 2–3 cm above the lateral knee joint line because the distal part of the iliotibial tract is inflamed (Fredericson and Weir 2006). In our case, the connection between the PM and the iliotibial tract was located around 4.5 cm above the knee joint line. In the cases described by Olewnik et al. (2020a), these connections were found 2–6 cm superior to the lateral knee joint line, so from an anatomical point of view, such a tendinous band between these two structures could affect the development of iliotibial band syndrome.

This comprehensive case report describes a newly-found variant of the PM and explores its potential clinical relevance. However, this is entirely an anatomical study that provides only purely morphological information. All suggested clinical implications of the presented PM are based on previous works relevant to the proximal attachment of the PM. Furthermore, many aspects of the PM remain unclear, and more multi-field examinations are needed. Nevertheless, we claim that by collecting all knowledge about the PM together, it is possible to define this muscle completely and understand its enigmatic features and possibilities.

## Conclusion

The plantaris muscle seems to be one of the most variable and mysterious human skeletal muscles. Its proximal attachment is as variable as its distal one. According to the current classification of plantaris muscle origins, the one described in this study is Type VI and is a new member of this ‘rare cases’ group. It is characterized by a bifurcated muscle belly (superior and inferior part), tendinous connection to the iliotibial tract and attachment to the semimembranosus tendon. Some of these features could lead potentially to clinical problems so it is suggested that clinicians should be aware of its existence and capacity to evolve. The plantaris muscle should be subjected to further extensive examinations.

## Data Availability

Please contact authors for data requests (Konrad Kurtys—e-mail: kurtyskonrad@gmail.com).
